# ﻿Three new species of *Mycena* sect. *Calodontes* (Mycenaceae, Agaricales) from Northeastern China

**DOI:** 10.3897/mycokeys.125.169722

**Published:** 2025-11-14

**Authors:** Jingwen Guo, Zewei Liu, Hui Zeng, Yupeng Ge, Qin Na

**Affiliations:** 1 School of Horticulture, Ludong University, Yantai 264025, China Ludong University Yantai China; 2 Institute of Edible Fungi, Fujian Academy of Agricultural Sciences; National and Local Joint Engineering Research Center for Breeding & Cultivation of Features Edible Fungi, Fuzhou 350014, China Institute of Edible Fungi, Fujian Academy of Agricultural Sciences Fuzhou China

**Keywords:** New taxon, systematics, taxonomy, white-spored fungi

## Abstract

Mycena sect. Calodontes is a large section within the genus Mycena, with 44 species described worldwide. The section is well characterized by relatively large basidiomata, typically growing on the humus layer of coniferous or coniferous-broadleaved mixed forests. Only 19 species of sect. Calodontes have been previously recorded in China, more than half of sect. Calodontes species are distributed in east and north regions, but also distributed in west and south regions. Based on 8 specimens collected from Heilongjiang and Jilin Province, 3 novel species are formally described: *M. brunneocystidiata***sp. nov.**, *M. roseopurpurea***sp. nov.**, and *M. rubrofusca***sp. nov.***Mycena brunneocystidiata* is distinguished by purple-brown to brown pileus, brown lamellae margins, and cystidia with brown contents. *Mycena roseopurpurea* is characterized by the pileus with brownish center, white lamellae margins, and colorless, fusiform cystidia. *Mycena rubrofusca* is distinguished by brownish pileus, white lamellae margins, and colorless, utriform cystidia. Detailed morphological descriptions, high-resolution habitat photographs, line drawings, and comparison with closely related taxa are provided for the new species. A combined phylogenetic analysis was conducted, based on a multi-locus (ITS+*RPB1*+*tef1*-*α*) dataset under Bayesian Inference (BI) and Maximum Likelihood (ML) analyses. The morphological data and the results of the phylogenetic analyses support the recognition of the 3 new species. A key to the 20 species currently known species of sect. Calodontes in China is also provided.

## ﻿Introduction

*Mycena* (Pers.) Roussel is a typical saprobic fungus which plays a pivotal role in forest ecosystems (Smith 1947; Perry 2002; Kirk et al. 2008; Na 2019). Species in this genus are known for their ability to decompose rotten branches and wood, fallen leaves, and various organic substrates, and could be facilitating nutrient cycling and sustaining energy flow (Fukasawa et al. 2009; Baldrian and Kohout 2017; Kyaschenko et al. 2017; Guerreiro et al. 2023). Within the genus, sect. Calodontes (Fr. Ex Berk.) Quél. is recognized for its large numbers of species and relatively large basidiomata, in which the pileus diameter can reach 6 cm, hygrophanous pileus, irregularly intervenose lamellae, mostly amyloid basidiospores, and smooth cystidia, pileipellis, and stipitipellis (Smith 1947; Maas Geesteranus 1992a, 1992b; Maas Geesteranus and de Meijer 1997; Grgurinovic 2003; Robich 2003; Aronsen and Læssøe 2016; Na 2019; Liu 2023). Mycena sect. Calodontes is primarily distributed in the mid- to high- latitude regions of the Northern Hemisphere, including Europe and America, with only a few species known from lower latitudes in the Southern Hemisphere (Maas Geesteranus 1992a, 1992b; Grgurinovic 2003; Chew et al. 2014; Cooper et al. 2018; Liu et al. 2021, 2022, 2024; Cortés-Pérez et al. 2023). In 1821, Fries erected subtri. *Calodontes* under Agaricus trib. Clitocybe, later Berkeley (1836) elevated *Clitocybe* (Fr.) Staude to subgenus rank, and sect. Calodontes was raised to the rank of section, and the concept of sect. Calodontes has been widely adopted (Fries 1821; Berkeley 1836; Quéltet 1875; Maas Geesteranus 1992a, 1992b; Grgurinovic 2003; Aronsen and Læssøe 2016; Na 2019; Liu 2023). By the early 21^st^ century, 44 species of sect. Calodontes are known, including 17 newly species recorded from Asia, 5 from North America, 2 from Africa, and 1 from Oceania (Grgurinovic 2003; Chew et al. 2014; Cooper et al. 2018; Liu et al. 2021, 2022, 2024; Qiang and Bai 2022; Cortés-Pérez et al. 2023; Fan et al. 2024; Nagamune et al. 2024; Xiao et al. 2025). Species of sect. Calodontes adapt to various temperature and humidity conditions, widely growing on the humus layer of coniferous forests in North Temperate and Cold Temperate Zones, a few species also reported from tropical and subtropical climates, including India, Malaysia, São Tomé, and southern Australia (Maas Geesteranus 1992a, 1992b; Grgurinovic 2003; Chew et al. 2014; Cooper et al. 2018; Cortés-Pérez et al. 2023; Liu 2023).

Over the past three decades, the color of pileus, and the shape, contents, thickness of the walls of cheilocystidia have been confirmed to be the diagnostic characters of sect. Calodontes (Maas Geesteranus 1992a, 1992b; Grgurinovic 2003; Robich 2003; Harder et al. 2010, 2013; Liu 2023). The color of pileus in sect. Calodontes, ranging from pink, sulfur yellow, white, purple to brown, is easily changed by the temperature, humidity, and growth stage, so the characteristics of cheilocystidia show more effectiveness (Kauserud et al. 2008; Liu 2023). In sect. Calodontes, cheilocystidia are fusiform, clavate, or utriform (sometimes with tapering apices), and apart from their shape, the thickness of their walls can also be used to distinguish some species from closely related taxa (Maas Geesteranus 1992a, 1992b; Grgurinovic 2003; Robich 2003; Liu et al. 2021, 2022; Liu 2023). According to a phylogenetic reconstruction of sect. Calodontes, based on the materials from Europe and North America, derived from an internal transcribed spacer (ITS), RNA polymerase II largest subunit (*RPB1*), and translation elongation factor-1 alpha (*tef1-α*) sequence dataset, the results supported the color of pileus and the characteristics of cheilocystidia can be used to delimit species, but the varieties and forms were not supported (Harder et al. 2010, 2013). Additionally, Harder et al. (2010, 2013) also proposed that the *RPB1* and *tef1*-*α* sequences improved the ability to identify phylogenetic species in the *M. pura* complex (Harder et al. 2010, 2013).

## ﻿Materials and methods

### ﻿Specimen collection and macroscopic characteristics recording

During field investigations, each specimen was assigned a unique collection number. High-resolution photos were taken using a Canon EOS 90D digital camera (Canon, Tokyo, Japan) equipped with an EF-S 60 mm f/2.8 Macro USM lens. Comprehensive ecological data were recorded, including forest type, substrate, elevation, climate, season, GPS coordinates, and macroscopic characteristics such as pileus, lamellae (intervenose), context, stipe (base), odor, and taste. Color codes and notations followed Ridgway (Ridgway 1912). A small fragment of context was excised from each specimen for molecular analyses after macroscopic documentation. Specimens were dried at 40 °C using a Stöckli dehydrator (A. & J. Stöckli AG, Netstal, Switzerland) and stored in self-sealing plastic bags containing color-changing silica gel. All voucher specimens were deposited in the Fungarium of the Fujian Academy of Agricultural Sciences (FFAAS), China.

### ﻿Microscopic characteristics’ observation and morphological description

Dried specimens were rehydrated in 5% KOH aqueous solution and examined using a Lab A1 light microscope (Carl Zeiss AG, Jena, Germany). The tissues were stained with 1% Congo red aqueous solution when necessary. Microscopic structures were photographed and measured using ZEN 2.3 software. For each specimen, basidiospores were observed in lateral view, and 20 mature basidiospores were randomly measured. The dimensions of basidiospores and Q values are presented as [a/b/c] (d)e–***f***–g(h) × (i)j–***k***–l(m) µm [Q = (n)o–p(q), Q_m_ = ***r*** ± s]. The notation [a/b/c] refers to the number of basidiospores, basidiomata, and specimens measured, respectively. Spore dimensions are presented as (d)e–***f***–g(h) × (i)j–***k***–l(m), where d and h denote the 5% minimum and maximum values, e–g indicate the central 90% range, and f represents the mean value. Q represents the length-to-width ratio, with Q = (n)o–p(q) indicating the range, and Q_m_ = ***r*** ± *s* denoting the mean and standard deviation. For the type specimen, two basidiomata were examined, with at least 20 mature basidiospores measured per individual, following Na et al. (2021), Liu et al. (2021, 2022), and Wei et al. (2024). Amyloid reactions of basidiospores and lamellar trama were tested using Melzer’s reagent (Vizzini et al. 2020). For all other microscopic structures, at least 20 basidia were measured, as well as measurements of the shape of cheilocystidia, pleurocystidia (if present), and caulocystidia (if present), and the hyphae of the pileipellis and stipitipellis were also measured, the contents, and wall thickness of cheilocystidia, pleurocystidia, and caulocystidia were also observed (Liu et al. 2022). Line drawings were prepared based on habitat photographs, field notes, and microscopic observations, scanned using a Canon LiDE120 scanner (Canon, Tokyo, Japan), and finalized using Adobe Photoshop 2023.

### ﻿Phylogenetic analyses

#### ﻿DNA sequence acquisition

Genomic DNA was extracted using the New Plant Genomic DNA Extraction Kit (Cowin Century, Beijing, China) and stored at -20 °C. Three nuclear loci, comprising the internal transcribed spacer (ITS), RNA polymerase II largest subunit (*RPB1*), and translation elongation factor-1 alpha (*tef1*-*α*), were amplified using the primer pairs ITS1/ITS4, *RPB1*Mp_f1/*RPB1*Mp_r1, *tEF*Mp_f2/*tEF*Mp_r2, respectively (Harder et al. 2010, 2013). PCR reactions were performed in 25 μL volumes, containing 12.5 μL of 2 × Utaq PCR MasterMix (ZomanBio, Beijing, China), 1 μL of each primer, 2 μL of DNA template, with ddH_2_O added to reach the final volume (Liu et al. 2022). The PCR protocol for the ITS region consisted of an initial denaturation at 94 °C for 4 min, followed by 34 cycles of 94 °C for 45 s, 52 °C for 45 s, and 72 °C for 1 min, a final extension of 72 °C for 10 min (Harder et al. 2010). The amplification protocol for the *RPB1* and *tef1*-*α* regions was as follows: 94 °C for 1 min, then 10 cycles of 94 °C for 35 s, 53 °C for 45 s, 72 °C for 45 s, and 25 cycles of 94 °C for 35 s, 56 °C for 45 s, 72 °C for 45 s, ending with a final extension of 72 °C for 10 min (Harder et al. 2013). PCR products were sequenced by the Beijing Genomics Institute (Beijing, China). The PCR products were cloned using the pBLUE-T Kit (Beijing Zoman Biotechnology Co., Beijing, China) to generate high-quality sequences.

#### ﻿Phylogenetic analyses

All newly generated sequences from the collected specimens were compared using BLAST in the NCBI database (https://www.ncbi.nlm.nih.gov/). Homologous sequences showing nucleotide identity greater than 90% were downloaded from GenBank (https://www.ncbi.nlm.nih.gov/genbank). *Mycena rubromarginata* (Fr.) P. Kumm was selected as the outgroup for phylogenetic analyses (Harder et al. 2010, 2013; Liu et al. 2022). Sequence alignment was performed using MAFFT v.7.110, with gaps treated as missing data. Peak profiles of the newly generated sequences were examined in BioEdit to detect insertion and deletion sites, and regions containing multiple copies were encoded using degenerate bases (Hall 1999; Katoh et al. 2002, 2019; Alzohairy 2011). Phylogenetic analyses were conducted using both Bayesian Inference (BI) and Maximum Likelihood (ML) methods in MrBayes v3.2.6 and raxmGUI 2.0.10 (Posada and Crandall 1998; Nylander 2004; Edler et al. 2021). The sequence matrix of 3 nuclear loci were divided into 6 partitions: ITS1, 5.8S, ITS2, *RPB1* exons, *tef1*-*α* exons, and the combined introns regions of *RPB1* and *tef1*-*α*. MCMC was run with 6 chains, and conducted sampling at intervals of 10,000 generations until the Average Deviation of Split Frequencies was below 0.01; the first 25% of trees were discarded as burn-in, using the ‘sump’ and ‘sumt burnin’ commands to generate the results (Ronquist and Huelsenbeck 2003; Ronquist et al. 2012). Tracer v.1.7.2 was used to evaluate the Effective Sample Size (ESS) and Average Potential Scale Reduction Factor (PSRF) values as indicators of Bayesian inference (BI) analysis (Rambaut et al. 2018; Fabreti and Höhna 2022). For the Maximum Likelihood (ML) analysis, default parameters in RAxML were used with 1,000 rapid bootstrap replicates to assess branch support (Edler et al. 2021). The resulting phylogenetic trees were visualized with FigTree v.1.4.3.

## ﻿Results

### ﻿Phylogenetic analyses

**Figure 1.**
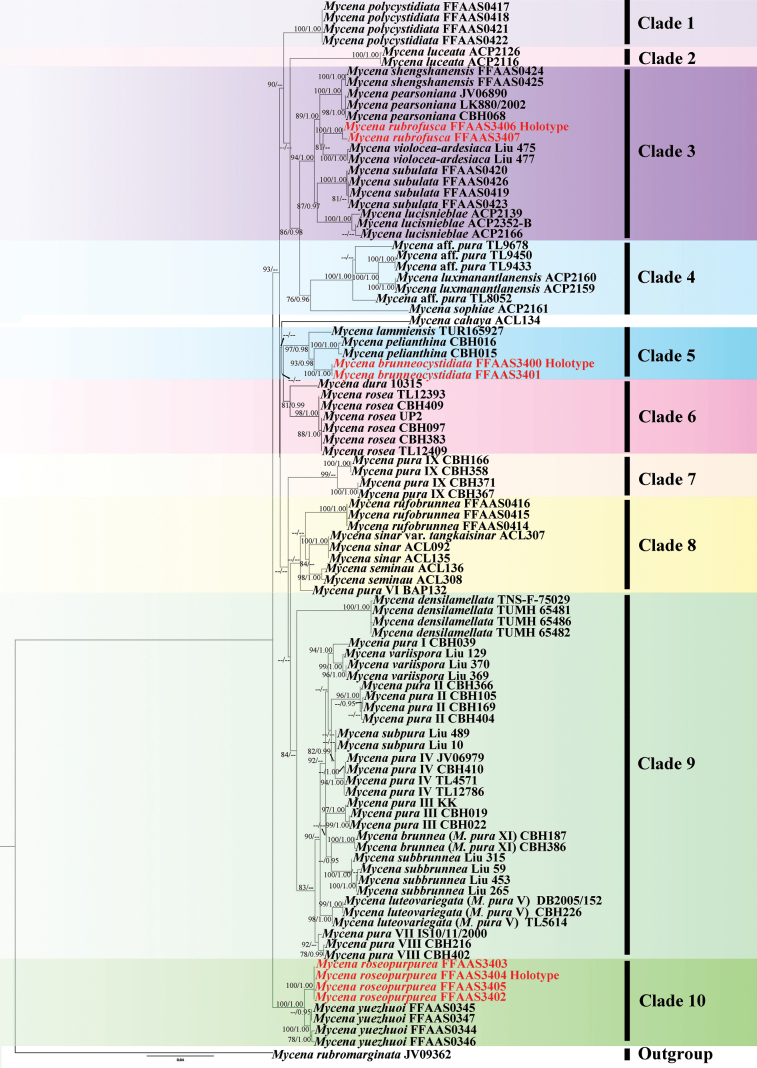
Bayesian Inference tree based on concatenated ITS+*RPB1*+*tef1*-*α* dataset. Only branch nodes with both Maximum Likelihood bootstrap support values (BS) above 75% and Bayesian posterior probabilities (BPP) exceeding 0.95 are indicated. New taxonomic groups are marked in red.

The dataset comprised 263 sequences, containing 24 newly generated sequences (8 ITS, 8 *RPB1*, and 8 *tef1*-*α*) and 239 sequences downloaded from GenBank (89 ITS, 70 *RPB1*, and 80 *tef1*-*α*). Detailed information for all sequences was provided in Table 1. The aligned dataset contained 1,538 nucleotide sites (including gaps), with 216 bp for ITS1 region, 159 bp for 5.8S region, 247 bp for ITS2 region, 55 bp for *RPB1* exons region, 307 bp for *tef1*-*α* exons region, 433 bp for *RPB1* introns region, and 121 bp for *tef1*-*α* introns region. Among the 24 newly generated sequences, 29 insertion sites and 23 deletion sites were identified, along with 2 degenerate bases, specifically R (1519 bp of *FFAAS3406*) and W (944 bp of *FFAAS3407*). For BI analysis, the best-fitting models for each partition of the concatenated dataset were selected as follows: GTR+G for ITS1 and *RPB1* introns+*tef1*-*α* introns, JC for 5.8S, GTR+I+G for ITS2 and *tef1*-*α* exons, and HKY for *RPB1* exons. The BI analysis, after 15,000,000 generations, yielded an average deviation of split frequencies of 0.006965, an effective sample size (ESS) of 1160.2, and a potential scale reduction factor (PSRF) ranging from 1.000 to 1.002. For ML analysis, the substitution models were as follows: JC for ITS1, 5.8S, and *RPB1* exons, K80+G for ITS2, TVMef+G for *tef1*-*α* exons, and HKY+G for *RPB1* and *tef1*-*α* introns regions. The final log-likelihood score was -8333.401114. The BI and ML analyses tree showed similar topologies, and the ML topology was selected to present the final phylogenetic tree (Fig. 1).

**Table 1.** Sequences of Mycena sect. Calodontes used in the phylogenetic analyses.

No.	Species	Voucher/Strain No.	GenBank NO.			Locality	Reference
			ITS	*RPB1*	*tef1-α*		
1	M. aff. pura	TL8052	FN394623	KF723687	KF723641	Ecuador	Harder et al. (2010, 2013)
2	M. aff. pura	TL9433	FN394622	KF723688	KF723642	Ecuador	Harder et al. (2010, 2013)
3	M. aff. pura	TL9450	KJ144653	KF723689	KF723643	Ecuador	Harder et al. (2010, 2013)
4	M. aff. pura	TL9678	FN394621	KF723690	KF723644	Ecuador	Harder et al. (2010, 2013)
5	*M. brunnea* (*M. pura* XI)	CBH187	FN394564	KF723678	KF723632	Denmark	Harder et al. (2010, 2013)
6	*M. brunnea* (*M. pura* XI)	CBH386	FN394565	KF723679	KF723633	Denmark	Harder et al. (2010, 2013)
7	***M. brunneocystidiata***	**FFAAS3400 Holotype**	**PV939239**	**PV952260**	**PV952232**	**China**	**This study**
8	***M. brunneocystidiata***	**FFAAS3401**	**PV939240**	**PV952261**	**PV952233**	**China**	**This study**
9	*M. cahaya*	ACL134	KF537248	–	–	Malaysia	Chew et al. (2014)
10	*M. densilamellata*	TUFC 101999	LC777686	LC777726	LC777734	Japan	Nagamune et al. (2024)
11	*M. densilamellata*	TUMH 65486	LC777687	LC777727	LC777735	Japan	Nagamune et al. (2024)
12	*M. densilamellata*	TUMH 65482	LC777688	LC777728	LC777736	Japan	Nagamune et al. (2024)
13	*M. densilamellata*	TNS-F-75029	LC777689	LC777729	LC777737	Japan	Nagamune et al. (2024)
14	*M. dura*	10315	FN394560	KF723694	KF723648	Austria	Harder et al. (2010, 2013)
15	*M. lammiensis*	TUR165927	FN394552	KF723697	KF723651	Finland	Harder et al. (2010, 2013)
16	*M. luceata*	ACP2116	OR233614	OR233746	OR233755	Mexico	Cortés-Pérez et al. (2023)
17	*M. luceata*	ACP2126	OR233613	OR233745	OR233754	Mexico	Cortés-Pérez et al. (2023)
18	*M. lucisnieblae*	ACP2139	OR233611	OR233743	OR233753	Mexico	Cortés-Pérez et al. (2023)
19	*M. lucisnieblae*	ACP2166	OR233607	OR233740	OR233750	Mexico	Cortés-Pérez et al. (2023)
20	*M. lucisnieblae*	ACP2352-B	OR233608	–	OR233756	Mexico	Cortés-Pérez et al. (2023)
21	*M. luteovariegata* (*M. pura* V)	CBH226	FN394604	KF723664	KF723618	Denmark	Harder et al. (2010, 2013)
22	*M. luteovariegata* (M. pura f. lutea)	DB2005/152	FN394603	–	–	Denmark	Harder et al. (2010)
23	*M. luteovariegata* (*M. pura* V)	TL5614	FN394602	KF723666	KF723620	Denmark	Harder et al. (2010, 2013)
24	*M. luxmanantlanensis*	ACP2160	OR233603	OR233737	OR233747	Mexico	Cortés-Pérez et al. (2023)
25	*M. luxmanantlanensis*	ACP2159	OR233604	OR233738	OR233748	Mexico	Cortés-Pérez et al. (2023)
26	*M. pearsoniana*	LK880/2002	FN394613	KF723693	KF723647	Germany	Harder et al. (2010, 2013)
27	*M. pearsoniana*	CBH068	FN394614	KF723691	KF723645	Germany	Harder et al. (2010, 2013)
28	*M. pearsoniana*	JV06890	FN394612	KF723692	KF723646	Denmark	Harder et al. (2010, 2013)
29	*M. pelianthina*	CBH015	FN394549	KF723695	KF723649	Denmark	Harder et al. (2010, 2013)
30	*M. pelianthina*	CBH016	FN394547	KF723696	KF723650	Denmark	Harder et al. (2010, 2013)
31	*M. polycystidiata*	FFAAS0417	ON427731	ON468456	ON468469	China	Liu et al. (2022)
32	*M. polycystidiata*	FFAAS0418	ON427732	ON468457	ON468470	China	Liu et al. (2022)
33	*M. polycystidiata*	FFAAS0421	ON427733	ON468458	ON468471	China	Liu et al. (2022)
34	*M. polycystidiata*	FFAAS0422	ON427734	ON468459	ON468472	China	Liu et al. (2022)
35	*M. pura* I	CBH039	FN394588	KF723680	KF723634	Denmark	Harder et al. (2010, 2013)
36	*M. pura* II	CBH105	FN394581	KF723671	KF723625	Denmark	Harder et al. (2010, 2013)
37	*M. pura* II	CBH169	FN394579	KF723672	KF723626	Denmark	Harder et al. (2010, 2013)
38	*M. pura* II	CBH366	FN394572	KF723673	KF723627	Denmark	Harder et al. (2010, 2013)
39	*M. pura* II	CBH404	FN394566	KF723674	KF723628	Denmark	Harder et al. (2010, 2013)
40	*M. pura* III	CBH019	FN394605	KF723675	KF723629	Denmark	Harder et al. (2010, 2013)
41	*M. pura* III	CBH022	FN394574	KF723676	KF723630	Denmark	Harder et al. (2010, 2013)
42	*M. pura* III	KK	FN394606	KF723677	KF723631	Slovakia	Harder et al. (2010, 2013)
43	*M. pura* IV	CBH410	FN394595	KF723667	KF723621	Denmark	Harder et al. (2010, 2013)
44	*M. pura* IV	JV06979	FN394585	KF723668	KF723622	Denmark	Harder et al. (2010, 2013)
45	*M. pura* IV	TL4571	FN394583	KF723669	KF723623	Denmark	Harder et al. (2010, 2013)
46	*M. pura* IV	TL12786	FN394591	KF723670	KF723624	Sweden	Harder et al. (2010, 2013)
47	*M. pura* VI	BAP132	FN394561	KF723660	KF723614	USA	Harder et al. (2010, 2013)
48	*M. pura* VII	IS10/11/2000	FN394611	–	–	USA	Harder et al. (2010)
49	*M. pura* VIII	CBH216	FN394598	KF723662	KF723616	Denmark	Harder et al. (2010, 2013)
50	*M. pura* VIII	CBH402	FN394599	KF723663	KF723617	Denmark	Harder et al. (2010, 2013)
51	*M. pura* IX	CBH166	FN394607	KF723701	KF723655	Denmark	Harder et al. (2010), 2013
52	*M. pura* IX	CBH358	FN394608	KF723702	KF723656	Denmark	Harder et al. (2010, 2013)
53	*M. pura* IX	CBH367	KF913022	KF723703	KF723657	Denmark	Harder et al. (2013)
54	*M. pura* IX	CBH371	KF913023	KF723704	KF723658	Denmark	Harder et al. (2013)
55	*M. rosea*	UP2	FN394550	–	–	UK	Harder et al. (2010)
56	*M. rosea*	CBH097	FN394556	KF723681	KF723635	Denmark	Harder et al. (2010, 2013)
57	*M. rosea*	CBH383	FN394553	KF723682	KF723636	Denmark	Harder et al. (2010, 2013)
58	*M. rosea*	CBH409	FN394551	KF723683	KF723637	Germany	Harder et al. (2010, 2013)
59	*M. rosea*	TL12393	FN394555	KF723684	KF723638	Denmark	Harder et al. (2010, 2013)
60	*M. rosea*	TL12409	FN394557	KF723685	KF723639	Denmark	Harder et al. (2010, 2013)
61	***M. roseopurpurea***	**FFAAS3402**	**PV939241**	**PV952262**	**PV952228**	**China**	**This study**
62	***M. roseopurpurea***	**FFAAS3403**	**PV939242**	**PV952263**	**PV952229**	**China**	**This study**
63	***M. roseopurpurea***	**FFAAS3404 Holotype**	**PV939243**	**PV952264**	**PV952230**	**China**	**This study**
64	***M. roseopurpurea***	**FFAAS3405**	**PV939244**	**PV952265**	**PV952231**	**China**	**This study**
65	***M. rubrofusca***	**FFAAS3406 Holotype**	**PV939245**	**PV952266**	**PV952235**	**China**	**This study**
66	***M. rubrofusca***	**FFAAS3407**	**PV939246**	**PV952267**	**PV952234**	**China**	**This study**
67	*M. rubromarginata*	JV09362	FN394624	KF723705	KF723659	Denmark	Harder et al. (2010, 2013)
68	*M. rufobrunnea*	FFAAS0414	ON427728	ON468453	ON468466	China	Liu et al. (2022)
69	*M. rufobrunnea*	FFAAS0415	ON427729	ON468454	ON468467	China	Liu et al. (2022)
70	*M. rufobrunnea*	FFAAS0416	ON427730	ON468455	ON468468	China	Liu et al. (2022)
71	*M. seminau*	ACL308	KF537252	–	–	Malaysia	Chew et al. (2014)
72	*M. seminau*	ACL136	KF537250	–	–	Malaysia	Chew et al. (2014)
73	*M. shengshanensis*	FFAAS0424	ON427739	ON468464	ON468477	China	Liu et al. (2022)
74	*M. shengshanensis*	FFAAS0425	ON427740	ON468465	ON468478	China	Liu et al. (2022)
75	*M. sinar*	ACL092	KF537247	–	–	Malaysia	Chew et al. (2014)
76	*M. sinar*	ACL135	KF537249	–	–	Malaysia	Chew et al. (2014)
77	M. sinar var. tangkaisinar	ACL307	KF537251	–	–	Malaysia	Chew et al. (2014)
78	*M. sophiae*	ACP2161	OR233605	–	OR233757	Mexico	Cortés-Pérez et al. (2023)
79	*M. subbrunnea*	Liu 59	PP037944	PP034075	PP034079	China	Liu et al. (2024)
80	*M. subbrunnea*	Liu 265	PP037946	PP034076	PP034081	China	Liu et al. (2024)
81	*M. subbrunnea*	Liu 315	PP037948	–	PP034083	China	Liu et al. (2024)
82	*M. subbrunnea*	Liu 453	PP037951	PP034077	PP034086	China	Liu et al. (2024)
83	*M. subpura*	Liu 10	PP037943	–	PP034078	China	Liu et al. (2024)
84	*M. subpura*	Liu 489	PP037954	–	PP034089	China	Liu et al. (2024)
85	*M. subulata*	FFAAS0419	ON427735	ON468460	ON468473	China	Liu et al. (2022)
86	*M. subulata*	FFAAS0420	ON427736	ON468461	ON468474	China	Liu et al. (2022)
87	*M. subulata*	FFAAS0423	ON427737	ON468462	ON468475	China	Liu et al. (2022)
88	*M. subulata*	FFAAS0426	ON427738	ON468463	ON468476	China	Liu et al. (2022)
89	*M. variispora*	Liu 129	PP037945	–	PP034080	China	Liu et al. (2024)
90	*M. variispora*	Liu 369	PP037949	–	PP034084	China	Liu et al. (2024)
91	*M. variispora*	Liu 370	PP037950	–	PP034085	China	Liu et al. (2024)
92	*M. violocea-ardesiaca*	Liu 475	PP037952	–	PP034087	China	Liu et al. (2024)
93	*M. violocea-ardesiaca*	Liu 477	PP037953	–	PP034088	China	Liu et al. (2024)
94	*M. yuezhuoi*	FFAAS0344	MW581490	MW868166	MW882249	China	Liu et al. (2021)
95	*M. yuezhuoi*	FFAAS0345	MW581491	MW868169	MW882250	China	Liu et al. (2021)
96	*M. yuezhuoi*	FFAAS0346	MW581492	MW868168	MW882251	China	Liu et al. (2021)
97	*M. yuezhuoi*	FFAAS0347	MW581493	MW868167	MW882252	China	Liu et al. (2021)

Remarks: New generated sequences are emphasized in bold; “-” show missing sequence.

According to the phylogenetic tree in Fig. 1 10 well-supported clades were identified. The three new species were located in Clade 3, Clade 5, and Clade 10, respectively, each forming an independent lineage with strong statistical support (BS/BPP = 100/1.00). Among the three clades, Clade 3 was morphologically characterized by the absence of pleurocystidia, and 6 species included in the clade: *M. rubrofusca*, *M. shengshanensis* Z.W. Liu, Y.P. Ge & Q. Na, *M. pearsoniana* Dennis ex Singer, *M. violocea-ardesiaca* Shun Liu & Biao Zhu, *M. subulata* Z.W. Liu, Y.P. Ge & Q. Na, and *M. lucisnieblae* Cortés-Pérez, Racm.-Cruz & Guzm.-Dáv. In Clade 3, *M. rubrofusca* and *M. violocea-ardesiaca* were identified as the two most closely related taxa but with low support (BS/BPP = 81/--). There were 3 species in Clade 5, *M. brunneocystidiata*, *M. pelianthina* (Fr.) Quél., and *M. lammiensis* Harmaja, the species in the clade have colored lamellae margins and cystidia with colored contents, *M. brunneocystidiata* showed a close phylogenetic relationship with *M. pelianthina* (BS/BPP = 93/0.98) than *M. lammiensis*. Clade 10 merely contained 2 species, *M. roseopurpurea* and *M. yuezhuoi* Z.W. Liu, Y.P. Ge & Q. Na, each formed a distinct lineage with statistical support (BS/BPP = 100/1.00). Furthermore, Clades 1, 2, 4, 6, 7, 8, and 9 also each formed separate lineages with high bootstrap and posterior probability values.

### ﻿Taxonomy

#### ﻿ Mycena brunneocystidiata

Taxon classificationFungiAgaricalesMycenaceae

J.W. Guo, Z.W. Liu, Y.P. Ge & Q. Na sp. nov.

9D5CF2E7-A99B-584C-95DD-68BE40CBA634

860072

Figs 2, 3, 4, 5

##### Diagnosis.

Pileus brown. Lamellae densely covered with dark brown dots, margin brown. Cheilocystidia, pleurocystidia, caulocystidia and terminal cells of stipitipellis with brownish contents. Differ from *M. lammiensis* by wider basidiospores (width > 4 μm) and fusiform caulocystidia.

**Figure 2.**
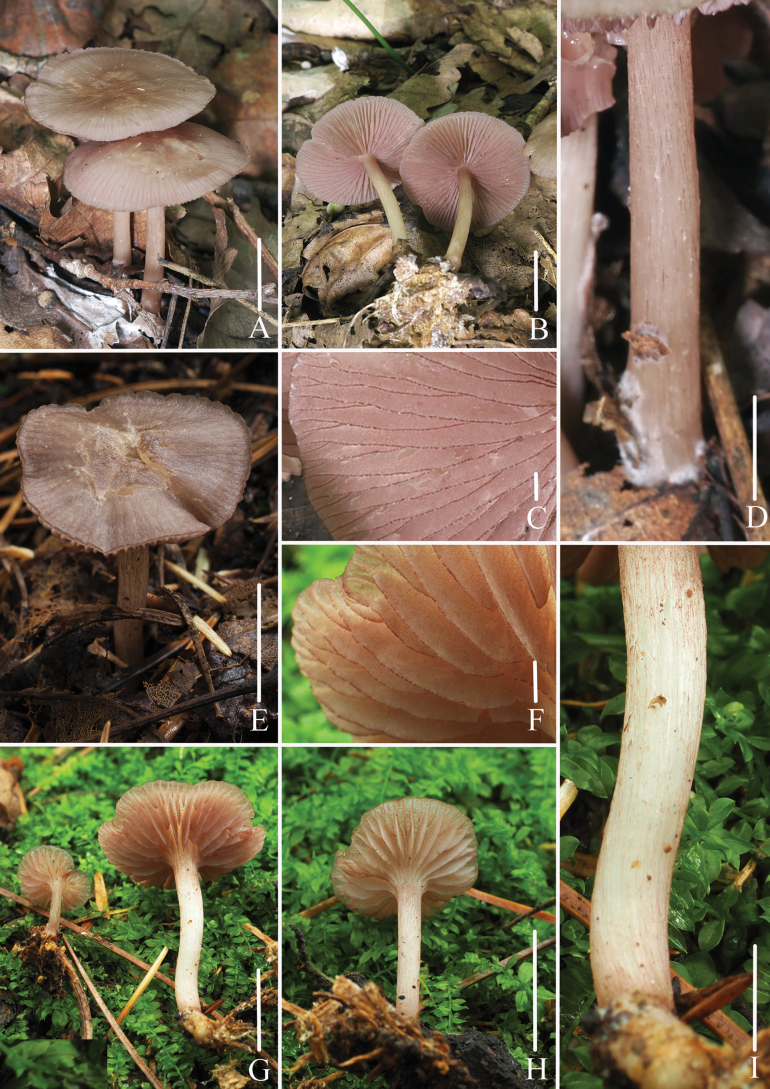
Basidiomata of *Mycena brunneocystidiata*. A–D. Collection *FFAAS3400*, holotype; E–I. Collection *FFAAS3401*. Scale bars: 20 mm (A–B, E, G–H); 1 mm (C, F); 5 mm (D, I). Photographs (A–I) by Jingwen Guo and Yupeng Ge.

##### Holotype.

China • Heilongjiang Province, Mudanjiang City, Mudanfeng National Forest Park, 42°45'74"N, 128°14'41"E, 22 August 2024, Jingwen Guo, Tian Wang, Qin Na, Zengcai Liu, Ruipeng Liu, Pengyu Du, Ying Yu, and Yupeng Ge leg., *FFAAS3400* (collection no. NJ 6538).

##### Etymology.

Name refers to the cheilocystidia, pleurocystidia, and caulocystidia with brown contents.

##### Description.

***Pileus*** 10–36 mm in diam., plano-convex, with slightly umbo at center, margin revolute, wavy, cracked at mature; *Drab (XLVI17′′′′) at center, gradually towards margin to PaMid Vinaceous-Drab (XLV5′′′′f), Pale Drab-Gray (XLVI17′′′′f) to *Drab-Gray (XLVI17′′′′d), margin *Drab (XLVI17′′′′); striate *Hair Brown (XLVI17′′′′i), towards the center up to 1/2–2/3 diam.. ***Context*** White (LIII), 1.0 mm thick, fragile. ***Lamellae*** subdecurrent, 23–27 reaching the stipe, 1–3 tiers of lamellulae, White (LIII), densely covered with Deep Brownish Drab (XLV9′′′′i) dots, irregularly intervenose, stretching downward to 2/3–3/4 of the width of lamellae, edge entirely Deep Brownish Drab (XLV9′′′′i), wavy. ***Stipe*** 24–44 × 2–4 mm, central, cylindrical; apex to middle *Smoke Gray (XLVI21′′′′d) to Pale Smoke Gray (XLVI21′′′′f), base *Ecru-Drab (XLVI13′′′′d) to *Drab-Gray (XLVI17′′′′d), hollow, fragile, apex with Light Drab (XLVI17′′′′b) to *Drab (XLVI17′′′′) striates, sparse White (LIII) pubescent at base. ***Odor*** and ***taste*** not distinctive.

**Figure 3.**
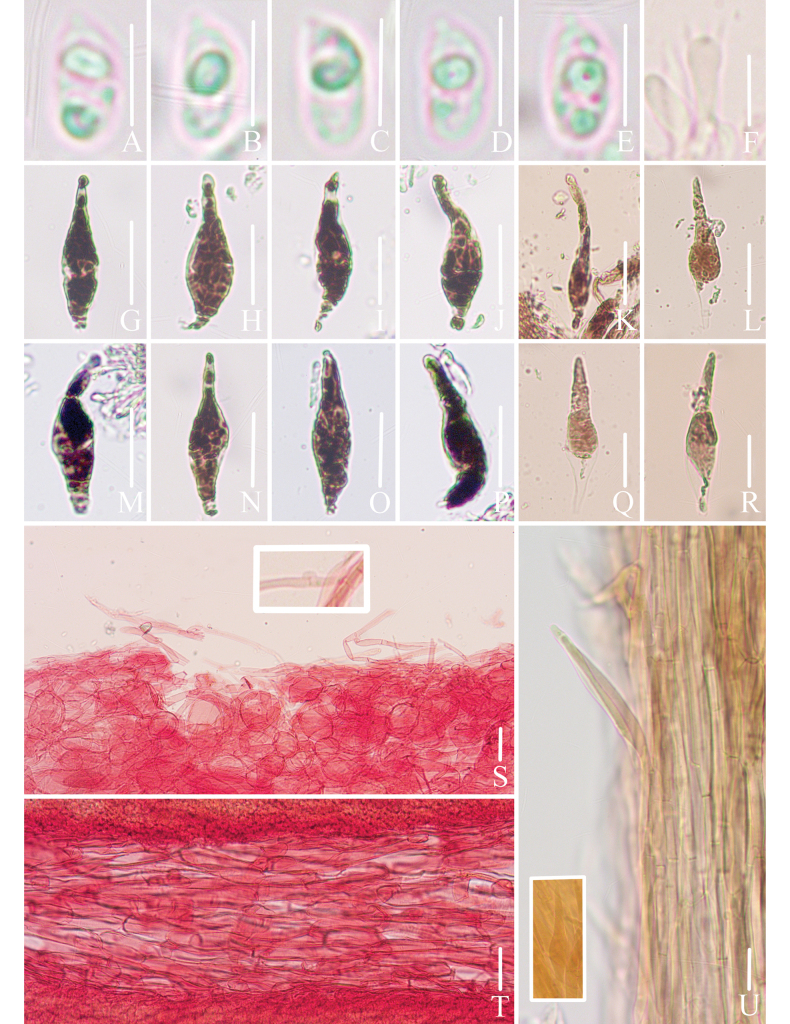
Microscopic features of *Mycena brunneocystidiata* (*FFAAS3400*, holotype). A–E. Basidiospores; F. Basidia; G–J. Fusiform cheilocystidia; K–L. Acicular to lanceolate cheilocystidia; M–P. Fusiform pleurocystidia; Q–R. Acicular to lanceolate pleurocystidia; S. Pileipellis and hypodermium; T. Lamellar trama; U. Stipitipellis and caulocystidia. Scale bars: 5 μm (A–E); 10 μm (F); 25 μm (G–R); 20 μm (S–U). Structures (A–E) were rehydrated in 5% KOH aqueous solution, (G–R, U) were rehydrated in sterile water and (F, S–T) were stained in 1% Congo red aqueous solution.

**Figure 4.**
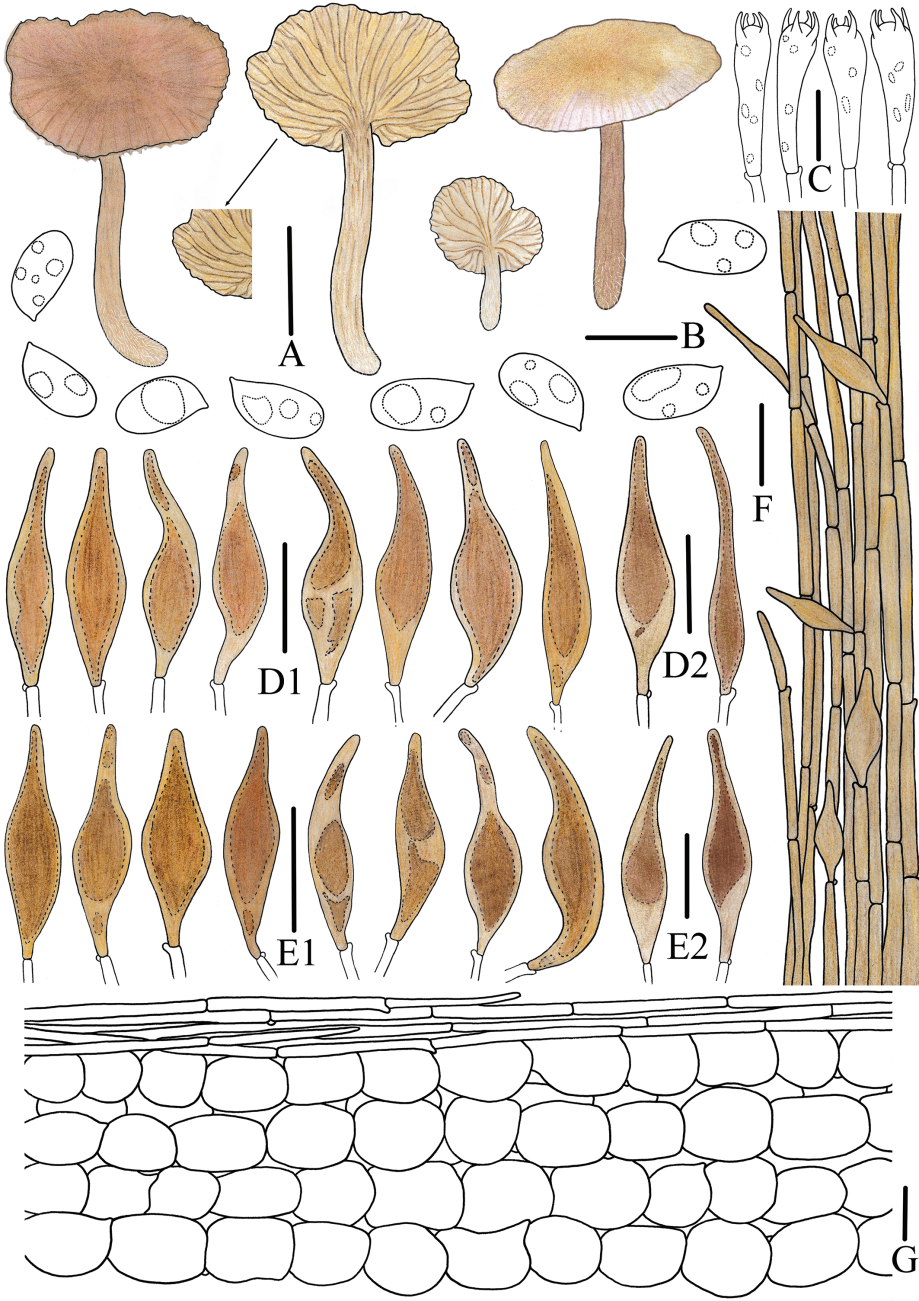
Morphological features of *Mycena brunneocystidiata* (*FFAAS3400*, holotype). A. Basidiomata; B. Basidiospores; C. Basidia; D1. Fusiform cheilocystidia; D2. Acicular to lanceolate cheilocystidia; E1. Fusiform pleurocystidia; E2. Acicular to lanceolate pleurocystidia; F. Stipitipellis and caulocystidia; G. Pileipellis and hypodermium. Scale bars: 20 mm (A); 5 μm (B); 10 μm (C); 25 μm (D1–G). Drawings by Jingwen Guo.

***Basidiospores*** (60/3/2) (5.4)5.6–***6.3***–6.9(7.1) × (2.6)2.9–***3.2***–3.5(4.0) μm [Q = (1.72)1.77–2.16(2.19), Q_m_ = ***1.97*** ± 0.09] [holotype (40/2/1) 6.0–***6.5***–6.9(7.1) × 3.0–***3.2***–3.5 μm [Q = (1.83)1.89–2.15(2.18), Q_m_ = ***2.00*** ± 0.08], narrowly ellipsoid to cylindrical, colorless, smooth (1000×), thin-walled, amyloid. ***Basidia*** clavate, 16–21 × 5–7 μm, hyaline, thin-walled, 4-spored, sterigmata 2–3 μm in length. ***Cheilocystidia*** fusiform with tapered apices, 36–62 × 9–14 μm (Fig. 3G–J, Fig. 4D1), acicular to lanceolate, 46–84 × 10–17 μm (Fig. 3M–P, Fig. 4D2), with brownish contents, thin-walled, smooth. ***Pleurocystidia*** similar to cheilocystidia, 39–72 × 8–15 μm, with brownish contents, thin-walled, smooth. ***Pileipellis*** a cutis composed of cylindrical cells, 32–121 × 3–6 μm, smooth, thin-walled; terminal cells cylindrical, apex tapering, 34–87 μm in length, apex 1–3 μm, base 3–7 μm, thin-walled, hyaline. ***Hypodermium*** formed by fusiform to subglobose hyphae, 20–72 × 12–39 μm, thin-walled, hyaline. ***Lamellar trama*** subregular, dextrinoid. ***Stipitipellis*** a cutis composed of cylindrical hyphae, 9–18 μm in diam., smooth, thin-walled; terminal cells fusiform or cylindrical, apex tapering, 31–72 × 4–8 μm, with pale brownish contents, thin-walled, smooth; ***caulocystidia*** fusiform, 33–70 × 7–13 μm, with pale brownish contents, thin-walled, smooth. ***Clamps*** present in all tissues.

##### Habit and habitat.

Scattered on the litter layer in *Acer mono* Maxim., *Larix gmelinii* (Ruprecht) Kuzeneva, *Pinus koraiensis* Siebold et Zuccarini, and *Quercus mongolica* Fischer ex Ledebour mixed forests during summer and autumn.

##### Known distribution.

Heilongjiang Province, Jilin Province, China.

##### Additional material examined.

China • Jilin Province, Yanbian Korean Autonomous Prefecture, Antu County, Erdaobaihe Town, Back Mountain of Changbai Mountain Natural History Museum, 42°46'43"N, 128°14'49"E, 17 August 2021, Zewei Liu, Qin Na, Shixin Wang, and Yupeng Ge leg., *FFAAS3401* (collection no. MY 0611).

##### Notes.

*Mycena brunneocystidiata* is considered to be a distinct species of sect. Calodontes subsect. Marginatae J. E. Lange on account of its lamellae margins brown and cheilocystidia, pleurocystidia, and caulocystidia with brown contents (Maas Geesteranus 1992a, 1992b). In the subsection, *M. lammiensis* and *M. pelianthina* share the same colored lamellae margins, cheilocystidia, and pleurocystidia with purple-brown contents, but *M. lammiensis* differs in having larger basidiospores (7.5–9.0 × 4.0–5.0 μm) and cylindrical caulocystidia, while *M. pelianthina* is identified by purple-brown pileus and lacking caulocystidia (Harmaja 1985; Robich 2003; Harder et al. 2010; Aronsen and Læssøe 2016). *Mycena shengshanensis* resembles *M. brunneocystidiata* in growing on the humus layer of *Larix gmelinii* and having light purple-brown to brown pileus, but differs in colorless, clavate, and thick-walled cheilocystidia (Liu et al. 2022).

**Figure 5.**
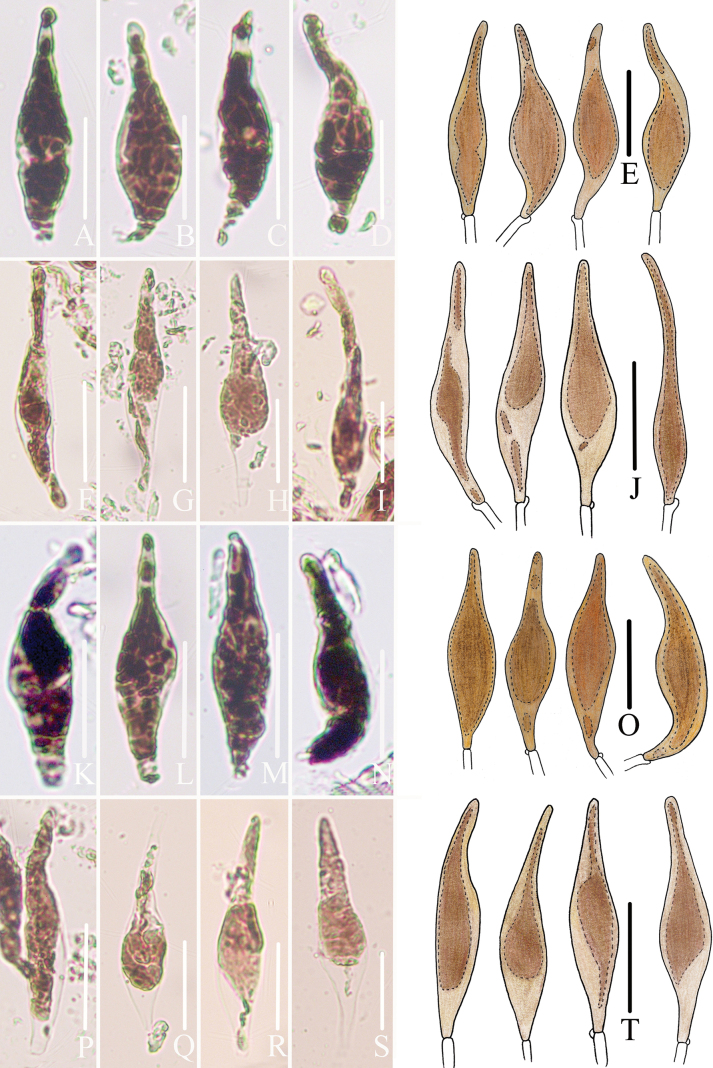
Morphological features of the Cheilocystidia and pleurocystidia of *Mycena brunneocystidiata*. A–E. Cheilocystidia of *FFAAS3401*; F–J. Cheilocystidia of *FFAAS3400*, holotype; K–O. Pleurocystidia of *FFAAS3401*; P–T. Pleurocystidia of *FFAAS3400*, holotype. Scale bars: 25 μm (A–E, K–O); 30 μm (F–J, P–T). Drawings by Jingwen Guo.

The main shape of cheilocystidia and pleurocystidia in *FFAAS3401* is fusiform with tapered apices (Fig. 5A–E, K–O), but in specimen *FFAAS3400*, acicular to lanceolate cheilocystidia and pleurocystidia can be observed occasionally, which are larger than the fusiform ones (Fig. 5F–J, P–T).

#### ﻿ Mycena roseopurpurea

Taxon classificationFungiAgaricalesMycenaceae

J.W. Guo, Z.W. Liu, Y.P. Ge & Q. Na sp. nov.

07E18D40-9E3A-5152-B03F-A94798B95305

860104

Figs 6, 7, 8

##### Diagnosis.

Pileus light pinkish-purple, light brown at center, hygrophanous when old. Cheilocystidia and pleurocystidia fusiform with tapered apices, thin-walled. Differ from *M. subulata* by lacking pleurocystidia and having acicular to lanceolate, thick-walled cheilocystidia.

**Figure 6.**
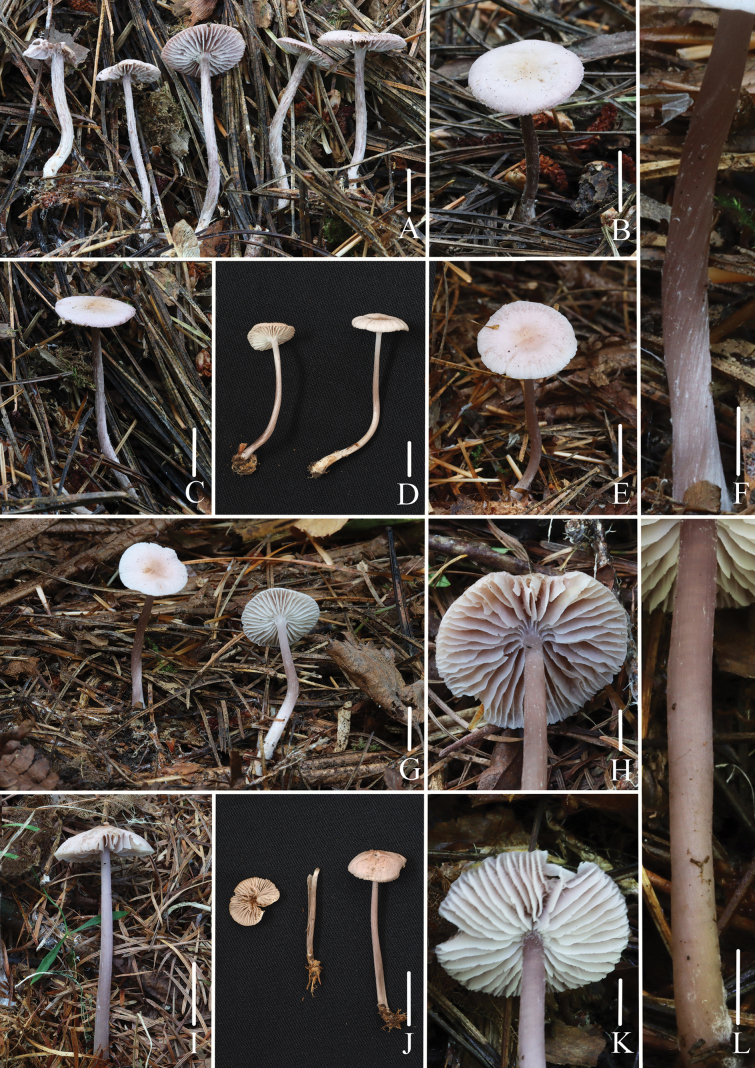
Basidiomata of *Mycena roseopurpurea*. A–C. Collection *FFAAS3405*; D–G. Collection *FFAAS3404*, holotype; H–J. Collection *FFAAS3403*; K–L. Collection *FFAAS3402*. Scale bars: 10 mm (A–E, G, I–J); 5 mm (F, H, K–L). Photographs (A–L) by Qin Na.

##### Holotype.

China • Heilongjiang Province, Yichun City, Liangshui National Nature Reserve, 47°12'74"N, 128°52'86"E, 21 August 2021, Zewei Liu, Qin Na, Shixin Wang, and Yupeng Ge leg., *FFAAS3404* (collection no. MY 0660).

**Figure 7.**
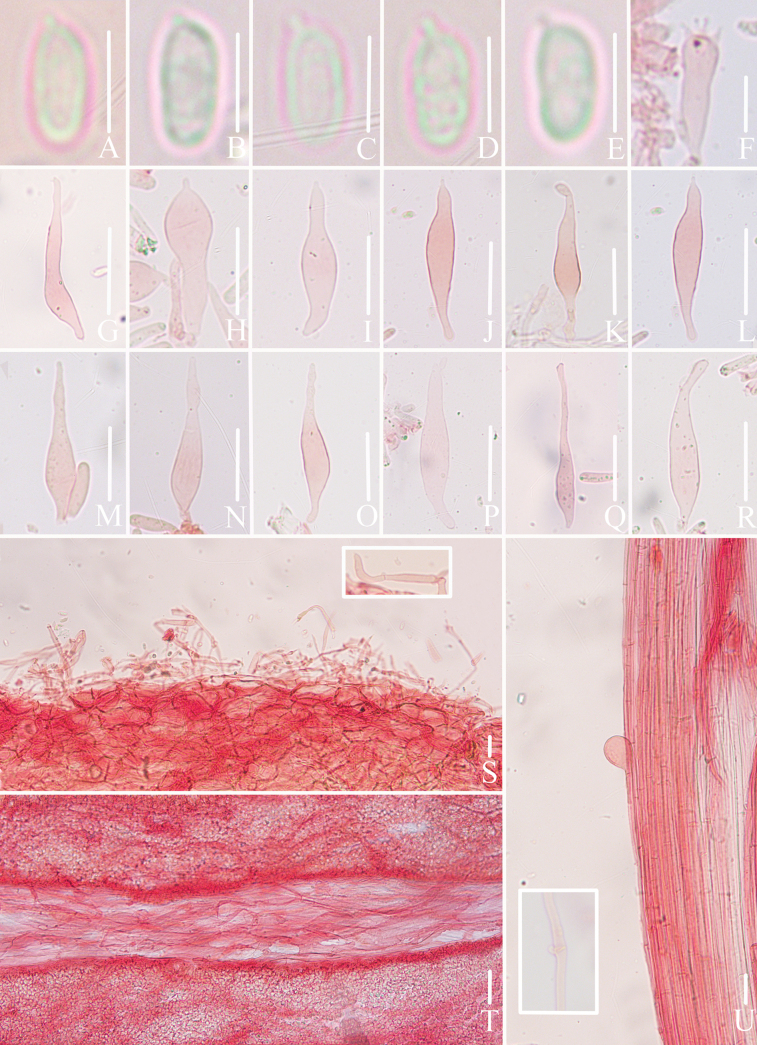
Microscopic features of *Mycena roseopurpurea* (*FFAAS3404*, holotype). A–E. Basidiospores; F. Basidia; G–L. Cheilocystidia; M–R. Pleurocystidia; S. Pileipellis and hypodermium; T. Lamellar trama; U. Stipitipellis and caulocystidia. Scale bars: 5 μm (A–E); 15 μm (F); 30 μm (G–U). Structures (A–E) were rehydrated in 5% KOH aqueous solution and (F–U) were stained in 1% Congo red aqueous solution.

##### Etymology.

Name refers to light pinkish-purple pileus.

**Figure 8.**
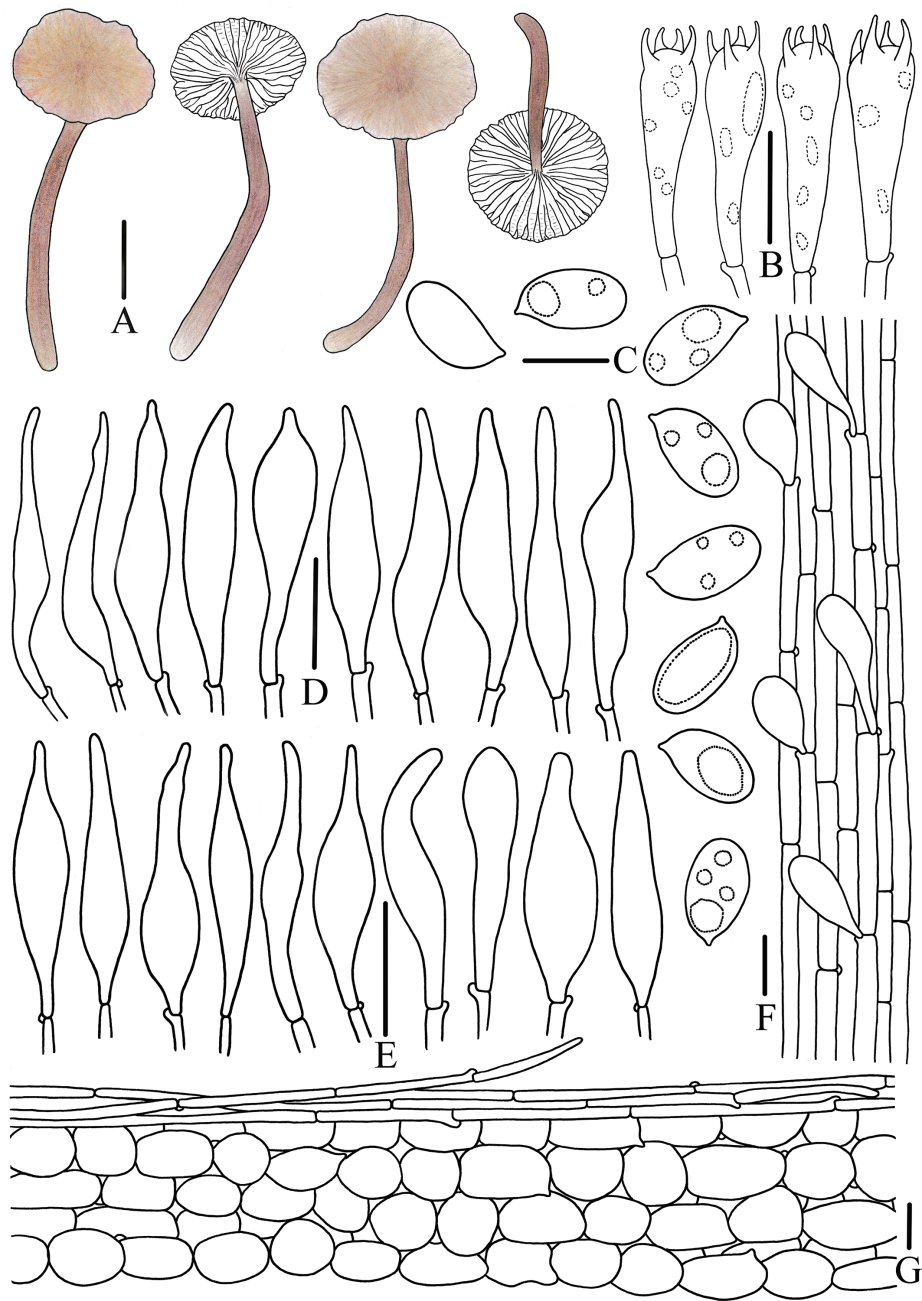
Morphological features of *Mycena roseopurpurea* (*FFAAS3404*, holotype). A. Basidiomata; B. Basidia; C. Basidiospores; D. Cheilocystidia; E. Pleurocystidia; F. Stipitipellis and caulocystidia; G. Pileipellis and hypodermium. Scale bars: 10 mm (A); 15 μm (B); 5 μm (C); 30 μm (D–G). Drawings by Jingwen Guo.

##### Description.

***Pileus*** 13–22 mm in diam., oblate hemispherical to plano-convex, with slightly depressed at center, margin wavy, occasionally cracked at mature; *Ecru-Drab (XLVI13′′′′d), Light Drab (XLVI17′′′′b) to *Drab-Gray (XLVI17′′′′d) at center, gradually towards margin to Pale Verbena Violet (XXXVI55′′f), Light Pinkish Lilac (XXXVII65′′f), Pale Grayish Vinaceous (XXXIX9′′′f), margin White (LIII); striate Pale Smoke Gray (XLVI21′′′′f), Pale Ecru-Drab (XLVI13′′′′f) to *Ecru-Drab (XLVI13′′′′d), towards the center up to 1/3 diam.; surface dry, hygrophanous when old. ***Context*** White (LIII), 1.0 mm thick, fragile. ***Lamellae*** sinuate to subdecurrent, 21–28 reaching the stipe, 1–3 tiers of lamellulae, White (LIII), irregularly intervenose, stretching downward to 1/3–3/4 of the width of lamellae, edge concolorous, wavy, serrated. ***Stipe*** 32–57 × 1–4 mm, central, cylindrical; apex Pale Drab-Gray (XLVI17′′′′f), Pale Vinaceous-Drab (XLV5′′′′d), Pale Brownish Drab (XLV9′′′′d), lower part Pale Purple-Drab (XLV1′′′′d), Light Purple-Drab (XLV1′′′′b) to *Ecru-Drab (XLVI13′′′′d), hollow, fragile; sparse White (LIII) pubescent at base. ***Odor*** and ***taste*** raphanoid.

***Basidiospores*** (100/5/4) (5.6)5.9–***6.7***–7.4(7.8) × 3.0–***3.5***–3.9(4.1) μm [Q = (1.70)1.72–2.10, Q_m_ = ***1.90*** ± 0.09] [holotype (40/2/1) (6.0)6.2–***6.7***–7.4(7.7) × 3.0–***3.5***–3.9(4.1) μm, Q = (1.71)1.77–2.12, Q_m_ = ***1.93*** ± 0.11], narrowly ellipsoid to cylindrical, colourless, smooth (1000×), thin-walled, amyloid. ***Basidia*** clavate, 18–31 × 4–7 μm, hyaline, thin-walled, 4-spored, sterigmata 2–4 μm in length. ***Cheilocystidia*** fusiform, subfusiform, with apices tapered, 35–97 × 7–18 μm, thin-walled, smooth. ***Pleurocystidia*** similar to cheilocystidia, 30–80 × 6–18 μm, thin-walled, smooth. ***Pileipellis*** a cutis composed of cylindrical cells, 29–81 × 3–11 μm, smooth, thin-walled; terminal cells cylindrical, apex tapering, 20–99 μm in length, apex 2–4 μm, base 2–9 μm, thin-walled, hyaline. ***Hypodermium*** formed by fusiform to subglobose hyphae, 20–76 × 6–37 μm, thin-walled, hyaline. ***Lamellar trama*** subregular, dextrinoid. ***Stipitipellis*** a cutis composed of cylindrical hyphae, 4–17 μm in diam., smooth, thin-walled; ***caulocystidia*** fusiform, clavate, sometimes utriform, 23–66 × 6–20 μm, thin-walled, smooth. ***Clamps*** present in all tissues.

##### Habit and habitat.

Scattered on the litter layers in *Betula platyphylla* Suk., *Larix gmelinii*, *Pinus koraiensis*, and *P. syluestriformis* (Takenouchi) T.Wang ex Cheng mixed forests during summer and autumn.

##### Known distribution.

Heilongjiang Province, Jilin Province, China.

##### Additional material examined.

China • Heilongjiang Province, Yichun City, Liangshui National Nature Reserve, 47°12'74"N, 128°52'56"E, 20 August 2021, Zewei Liu, Qin Na, Shixin Wang, and Yupeng Ge leg., *FFAAS3403* (collection no. MY 0635); • same location, 21 August 2021, Zewei Liu, Qin Na, Shixin Wang, and Yupeng Ge leg., *FFAAS3405* (collection no. MY 0668). • Jilin Province, Yanbian Korean Autonomous Prefecture, Antu County, Erdaobaihe Town Beauty Pine Forest, 42°45'74"N, 128°14'41"E, 18 August 2021, Zewei Liu, Qin Na, Shixin Wang, and Yupeng Ge leg., *FFAAS3402* (collection no. MY 0625).

##### Notes.

*Mycena subulata*, originally described from Heilongjiang province in China, can be easily mistaken for *M. roseopurpurea* due to morphological similarity, and growing on the humus layer of mixed forests of *Larix gmelinii* and *Pinus koraiensis*, but *M. subulata* is distinguished by acicular to lanceolate and thick-walled cheilocystidia (Liu et al. 2022). *Mycena pearsoniana*, while having similar stipe color and lacking pleurocystidia, differs by subfusiform or clavate cheilocystidia and inamyloid basidiospores (Smith 1947; Dennis 1970; Kost 2002; Harder et al. 2012; Aronsen and Læssøe 2016; Na 2019; Kwon et al. 2020). *Mycena dura* Maas Geesteranus & Hauskn. and *M. subcorticalis* (Cooke & Massee) Sacc., reported from Europe and southern Australia, resemble *M. roseopurpurea*; however, *M. dura* is distinctively characterized by its growth in grasslands and white stipe, and *M. subcorticalis* is distinguished by inamyloid basidiospores and gelatinized pileipellis (Saccardo 1891; Maas Geesteranus and Hausknecht 1994; Grgurinovic 2003; Olariaga et al. 2015). *Mycena pura* differs by having light purple to purplish-red pileus and pinkish-purple to purple stipe (Maas Geesteranus 1992a, 1992b; Perry 2002; Robich 2003; Thormann et al. 2006; Aronsen and Læssøe 2016; Na 2019; Okon et al. 2022; Liu 2023).

#### ﻿ Mycena rubrofusca

Taxon classificationFungiAgaricalesMycenaceae

J.W. Guo, Z.W. Liu, Y.P. Ge & Q. Na, sp. nov.,

FBB06453-D7BB-5059-B93C-E1AA68BD7753

860105

Figs 9, 10, 11

##### Diagnosis.

Pileus light reddish-brown to light grayish-brown, near margin hygrophanous. Pleurocystidia and caulocystidia absent. Differ from *M. polycystidiata* Z.W. Liu, Y.P. Ge, L. Zou & Q. Na by having pleurocystidia and caulocystidia.

**Figure 9.**
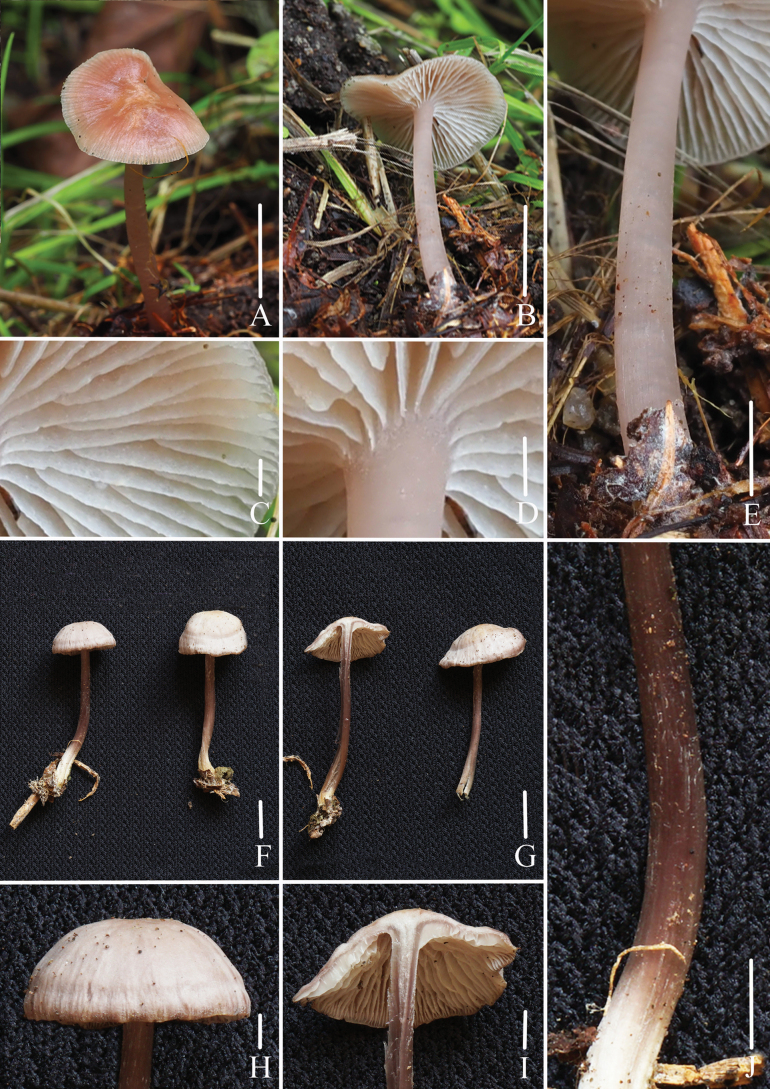
Basidiomata of *Mycena rubrofusca*. A–E. Collection *FFAAS3406*, holotype; F–J. Collection *FFAAS3407*. Scale bars: 15 mm (A–B, F–G); 1 mm (C–D); 5 mm (E, H–J). Photographs (A–J) by Jingwen Guo and Yupeng Ge.

##### Holotype.

China • Heilongjiang Province, Mudanjiang City, Mudanfeng National Forest Park, 42°45'74"N, 128°14'41"E, 22 August 2024, Jingwen Guo, Tian Wang, Qin Na, Zengcai Liu, Ruipeng Liu, Pengyu Du, Ying Yu, and Yupeng Ge leg., *FFAAS3406* (collection no. NJ 6508).

**Figure 10.**
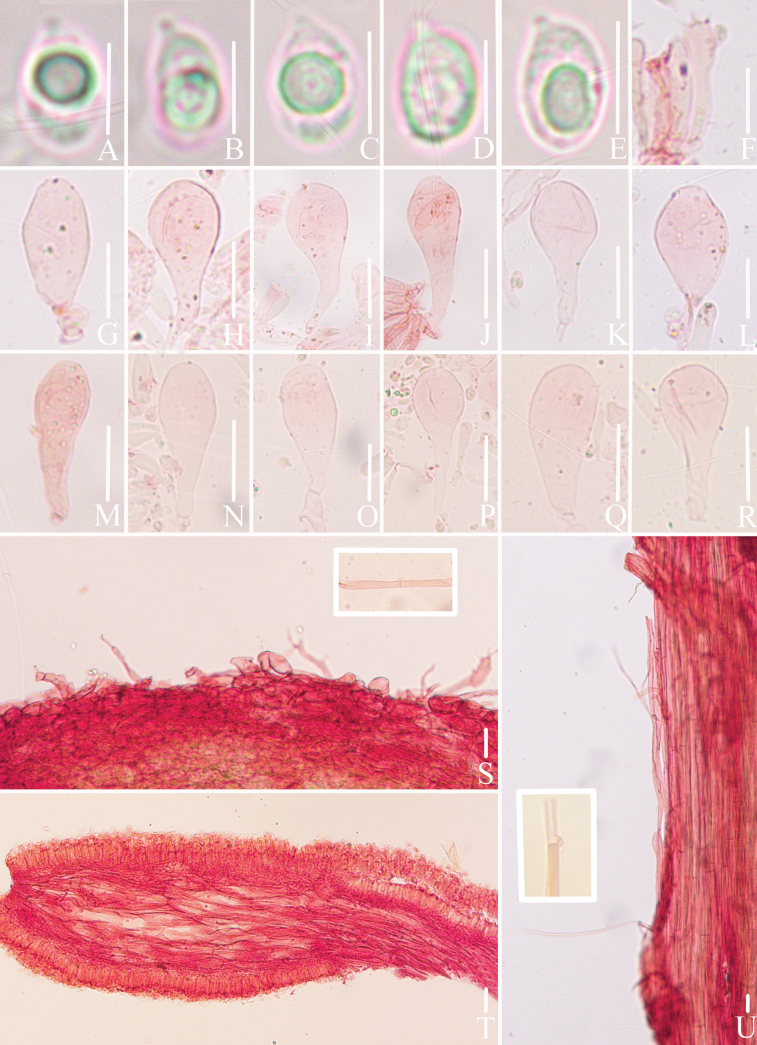
Microscopic features of *Mycena rubrofusca* (*FFAAS3406*, holotype). A–E. Basidiospores; F. Basidia; G–R. Cheilocystidia; S. Pileipellis and hypodermium; T. Lamellar trama; U. Stipitipellis. Bars: 5 μm (A–E); 15 μm (F, T, U); 25 μm (G–R); 30 μm (S). Structures (A–E) were rehydrated in 5% KOH aqueous solution and (F–U) were stained in 1% Congo red aqueous solution.

##### Etymology.

Name refers to the light reddish-brown, light grayish brown to brown pileus.

**Figure 11.**
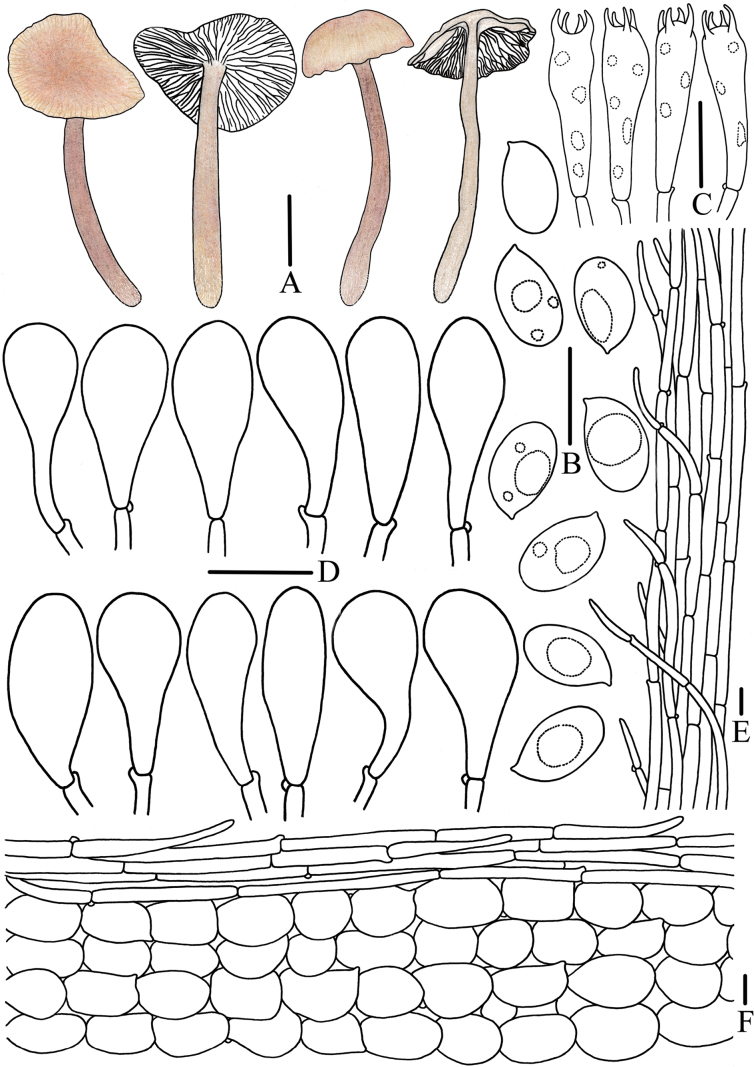
Morphological features of *Mycena rubrofusca* (*FFAAS3406*, holotype). A. Basidiomata; B. Basidiospores; C. Basidia; D. Cheilocystidia; E. Stipitipellis; F. Pileipellis and hypodermium. Scale bars: 15 mm (A, C, E–F); 5 μm (B); 25 μm (D). Drawings by Jingwen Guo.

##### Description.

***Pileus*** 17–26 mm in diam., hemispherical, plano-convex at mature, margin wavy; Light Drab (XLVI17′′′′b) at center, gradually towards margin to Light Vinaceous-Fawn (XL13′′′d), Pale Ecru-Drab (XLVI13′′′′f) to *Drab-Gray (XLVI17′′′′d), margin Light Drab (XLVI17′′′′b) to *Drab (XLVI17′′′′); striate *Drab (XLVI17′′′′), towards the center up to 1/8–1/7 diam.; surface moist, near margin hygrophanous. ***Context*** White (LIII), 1.0 mm thick, fragile. ***Lamellae*** adnexed or subdecurrent, approximately 38 reaching the stipe, 1–3 tiers of lamellulae, White (LIII), irregularly intervenose, stretching downward to 2/3–3/4 of the width of lamellae, edge concolorous, wavy. ***Stipe*** 44–45 × 2–4 mm, central, cylindrical; apex to lower part Vinaceous-Drab (XLV5′′′′), Light Brownish Drab (XLV9′′′′b) to *Hair brown (XLVI17′′′′i), base Light Grayish Olive (XLVI21′′′′b), hollow, fragile; base swollen, sparse White (LIII) pubescent at base. ***Odor*** and ***taste*** faintly raphanoid.

***Basidiospores*** (40/2/2) (5.1)6.2–***7.4***–8.5(8.8) × (3.8)4.0–***4.6***–5.5(5.7) μm [Q = (1.52)1.55–1.84(1.89), Q_m_ = ***1.69*** ± 0.10] [holotype (20/1/1) (6.8)7.2–***7.6***–8.3(8.5) × (3.8)4.1–***4.6***–4.8(5.0) μm [Q = 1.60–1.81, Q_m_ = ***1.70*** ± 0.08], ellipsoid to narrowly ellipsoid, colourless, smooth (1000×), thin-walled, amyloid. ***Basidia*** clavate, 16–28 × 5–8 μm, hyaline, thin-walled, 4-spored, sterigmata 2–4 μm in length. ***Cheilocystidia*** utriform, clavate, 24–67 × 8–23 μm, thin-walled, smooth. ***Pleurocystidia*** absent. ***Pileipellis*** a cutis composed of cylindrical cells, 28–76 × 5–15 μm, smooth, thin-walled; terminal cells cylindrical, apex tapering, 33–75 μm in length, apex 2–9 μm, base 3–11 μm, thin-walled, hyaline. ***Hypodermium*** formed by fusiform to subglobose hyphae, 26–84 × 11–34 μm, thin-walled, hyaline. ***Lamellar trama*** subregular, dextrinoid. ***Stipitipellis*** a cutis composed of cylindrical hyphae, 6–16 μm in diam, smooth, thin-walled; projecting terminal cells cylindrical or fusiform, 31–73 × 4–9 μm, thin-walled, smooth, hyaline; ***caulocystidia*** absent. ***Clamps*** present in all tissues.

##### Habit and habitat.

Scattered on the litter layers in *Fraxinus mandschurica* Rupr., *Pinus koraiensis*, and *Tilia amurensis* Rupr. mixed forests during summer and autumn.

##### Known distribution.

Heilongjiang Province, China.

##### Additional material examined.

China • Heilongjiang Province, Hegang City, Taipinggou National Nature Reserve, 48°12'43"N, 130°66'04"E, 3 September 2021, Zewei Liu, Qin Na, Shixin Wang, and Yupeng Ge leg., *FFAAS3407* (collection no. MY 0793).

##### Notes.

*Mycena polycystidiata* is the closest species to *M. rubrofusca* because it has a light grayish-brown to brown pileus and stipe, and utriform cheilocystidia, but *M. polycystidiata* has pleurocystidia and caulocystidia (Liu et al. 2022). *Mycena pura*, a widely distributed species in the North temperate zone, differs from *M. rubrofusca* by its fusiform, clavate, or subglobose cheilocystidia and pleurocystidia, and clavate or conical caulocystidia (Maas Geesteranus 1992a, 1992b; Perry 2002; Robich 2003; Thormann et al. 2006; Aronsen and Læssøe 2016; Na 2019; Okon et al. 2022; Liu 2023). Due to the light gray-brown to light brown pileus, *M. variispora* Shun Liu & Biao Zhu and *M. subbrunnea* Shun Liu & Biao Zhu are difficult to distinguish from *M. rubrofusca*, but they can be distinguished by the occurrence in mixed forests of *Larix gmelinii* and *Betula platyphylla*, purple lamellae, and clavate, thick-walled cheilocystidia (Liu et al. 2024). *Mycena pearsoniana* differs from *M. rubrofusca* in having purple pileus and clavate or fusiform caulocystidia (Smith 1947; Dennis 1970; Kost 2002; Harder et al. 2012; Aronsen and Læssøe 2016; Na 2019; Kwon et al. 2020). *Mycena rufobrunnea* Z.W. Liu, Y.P. Ge & Q. Na and *M. vinacea* Cleland, which were reported from northeastern China and southern Australia, respectively, both resemble *M. rubrofusca* in having utriform cheilocystidia (Cleland 1931; Liu et al. 2022). However, both of them also have clavate cheilocystidia; *M. rufobrunnea* is distinguished by a light brown to reddish-brown pileus, whereas *M. vinacea* is characterized by cylindrical pleurocystidia and basidiospores (Cleland 1931; Liu et al. 2022).

### ﻿Key to 20 species belonging to Mycena sect. Calodontes in China

**Table:** 

1	Lamellae edge purplish-brown or brown	**2**
–	Lamellae edge white	**3**
2	Caulocystidia present	***M. brunneocystidiata***
–	Caulocystidia absent	***M. pelianthina***
3	Pileus pink	***M. rosea***
–	Pileus not pink	**4**
4	Cheilocystidia thick-walled	**5**
–	Cheilocystidia thin-walled	**10**
5	Pileus lilac	***M. variispora***
–	Pileus not lilac	**6**
6	Lamellae surfaces white	**7**
–	Lamellae surfaces lilac	**9**
7	Cheilocystidia acicular to lanceolate	***M. subulata***
–	Cheilocystidia clavate	**8**
8	Stipe light grayish-brown to purplish-brown	***M. shengshanensis***
–	Stipe white to pinkish-yellow	***M. subbrunnea***
9	Lamellae adnexed	***M. subpura***
–	Lamellae subfree	***M. violocea-ardesiaca***
10	Basidiospores inamyloid	**11**
–	Basidiospores amyloid	**12**
11	Pileus white to cream-colored	***M. glabera***
–	Pileus pinkish-purple to purple	***M. pearsoniana***
12	Cheilocystidia utriform	**13**
–	Cheilocystidia fusiform or clavate	**15**
13	Pleurocystidia present	***M. polycystidiata***
–	Pleurocystidia absent	**14**
14	Caulocystidia present	***M. rufobrunnea***
–	Caulocystidia absent	***M. rubrofusca***
15	Pileus white	**16**
–	Pileus not white	**17**
16	Lamellae adnate	***M. cinereoalba***
–	Lamellae adnexed	***M. subaquosa***
17	Pleurocystidia present	**18**
–	Pleurocystidia absent	***M. yuezhuoi***
18	Stipe purple	***M. pura***
–	Stipe not purple	**19**
19	Basidiospores (5.6)5.9–7.4(7.8) × 3.0–3.9(4.1) μm, elongated ellipsoid to cylindrical	***M. roseopurpurea***
–	Basidiospores (8.3)8.6–10.8(11.5) × (5.3)5.4–6.3(6.4) μm, ellipsoid to elongated ellipsoid	***M. roseolamellata***

## ﻿Discussion

The comprehensive morphological characteristics of sect. Calodontes cannot be currently shown by the taxonomic systems (Harder et al. 2010, 2013; Liu 2023). Three subsections were proposed based on the amyloid reaction of basidiospores, the presence of colored contents in cheilocystidia and pleurocystidia, and the presence of pleurocystidia, namely subsect. Purae Konrad & Maubl., subsect. Marginatae, and subsect. Violacellae Singer ex Maas Geest. (Maas Geesteranus 1992a, 1992b). In this study, *M. brunneocystidiata* was assigned to subsect. Marginatae due to its amyloid basidiospores and cheilocystidia, pleurocystidia, and caulocystidia with brown contents; *M. roseopurpurea* was assigned to subsect. Purae owing to its amyloid basidiospores and having colorless cheilocystidia and pleurocystidia. However, *M. rubrofusca*, which was assigned to subsect. Violacellae due to its lack of pleurocystidia, cannot be placed in this subsection on account of its amyloid basidiospores. Notably, Harder et al. (2012) and Pérez-De-Gregorio (2024) reported that *M. pearsoniana* was assigned to subsect. Violacellae, which was defined by Maas Geesteranus based on inamyloid basidiospores, but its basidiospores showed inamyloid to weakly amyloid basidiospores after 40 minutes (Maas Geesteranus 1992a, 1992b; Harder et al. 2012; Pérez-De-Gregorio 2024). The subsections and species were also not supported by the multi-locus (ITS+*RPB1*+*tef1*-*α*) analyses, subsect. Marginatae was monophyletic, and subsect. Purae and subsect. Violacellae were polyphyletic (Harder et al. 2010, 2012, 2013; Chew et al. 2014; Liu 2023). Species clustered into a single clade with high support in phylogenetic analyses showed significant morphological differences. *Mycena subbrunnea*, *M. variispora*, *M. subpura* Shun Liu & Biao Zhu, and 7 phylogenetic species from the *M. pura* complex were gathered in the same clade, but the shape and wall thickness of cheilocystidia, and the presence or absence of pleurocystidia were different (Maas Geesteranus 1992a, 1992b; Perry 2002; Robich 2003; Thormann et al. 2006; Harder et al. 2010, 2013; Liu 2023; Liu et al. 2024).

According to previous literature, species of sect. Calodontes predominantly inhabit coniferous forests or coniferous-broadleaved mixed forests in Europe, North America, Asia, and Northern Africa, mainly within the Northern Hemisphere (Maas Geesteranus 1992a, 1992b; Robich 2003; Harder et al. 2010, 2013; Chew et al. 2014; Aronsen and Læssøe 2016; Cooper et al. 2018; Cortés-Pérez et al. 2023). Only a few occur in grasslands or in broadleaved forests of the Southern Hemisphere, southern Oceania (Grgurinovic 2003). In China, species of sect. Calodontes are predominantly found in the litter layer of coniferous-broadleaved mixed forests in Northeast China, and in coniferous forests in East, Southwest, Northwest, and Southeast China (Bau et al. 2019; Na 2019; Liu et al. 2021, 2022, 2024; Liu 2023; Fan et al. 2024; Xiao et al. 2025). Most species of sect. Calodontes, including the three new species in this study, are distributed in low-altitude regions (< 1000 m), with a few recorded in mid-altitude areas (1000–3500 m), and no species have been reported from high-altitude regions (3500–5000 m) (Maas Geesteranus 1992a, 1992b; Maas Geesteranus and de Meijer 1997; Grgurinovic 2003; Cooper et al. 2018; Bau et al. 2019; Na 2019; Liu 2023; Gao et al. 2024).

## Supplementary Material

XML Treatment for Mycena brunneocystidiata

XML Treatment for Mycena roseopurpurea

XML Treatment for Mycena rubrofusca

## ﻿Additional information

### Use of AI

No use of AI was reported.

### Author ORCIDs

Jingwen Guo https://orcid.org/0009-0005-2779-7456

Zewei Liu https://orcid.org/0000-0002-2700-0831

Hui Zeng https://orcid.org/0000-0003-2025-844X

Yupeng Ge https://orcid.org/0000-0001-5754-201X

Qin Na https://orcid.org/0000-0001-8406-6389

### Data availability

All of the data that support the findings of this study are available in the main text or Supplementary Information.

## Supplementary materials

Supplementary material 1 Fasta of Phylogentic tree Data type fas https://binary.pensoft.net/file/1465967This dataset is made available under the Open Database License (http://opendatacommons.org/licenses/odbl/1.0/). The Open Database License (ODbL) is a license agreement intended to allow users to freely share, modify, and use this Dataset while maintaining this same freedom for others, provided that the original source and author(s) are credited.Jingwen Guo, Zewei Liu, Hui Zeng, Yupeng Ge, Qin Na
